# Hydrophobic Hydration and Light Transport in α-Synuclein Protein Solutions in the Near-Infrared

**DOI:** 10.1177/00037028251367004

**Published:** 2025-07-30

**Authors:** Marco A. Saraiva

**Affiliations:** 1 Centro de Química Estrutural, Institute of Molecular Sciences, 72971 Instituto Superior Técnico, University of Lisbon, 1049-001 Lisbon, Portugal; 2 98819 Instituto de Tecnologia Química e Biológica António Xavier, Universidade Nova de Lisboa, Av. da República, 2780-157 Oeiras, Portugal

**Keywords:** α-Synuclein, near-infrared light, NIR, hydrophobic hydration, protein aggregation

## Abstract

Currently, there is increasing interest in identifying the mechanistic characteristics of the α-synuclein amyloid protein aggregation during its early stages. The initiation of amyloid protein incubation was investigated by applying the concepts of hydrophobic hydration in the early-formed protein aggregates and the light transport in the protein samples by using near-infrared light. These are unexplored concepts in amyloid protein aggregation research. Early-formed protein aggregates develop solvent-exposed hydrophobic residue segments, and intramolecular and intermolecular interactions can be identified by hydrophobic hydration, while consecutive intramolecular interactions can cancel this effect. In the light transport within protein samples, at low protein concentrations, the early-formed protein aggregates achieve stability, whereas at higher concentrations, such as those found in neuronal synapses (∼50  µM), the early-formed aggregates continue to develop.

## Graphical abstract

**Graphical abstract:**
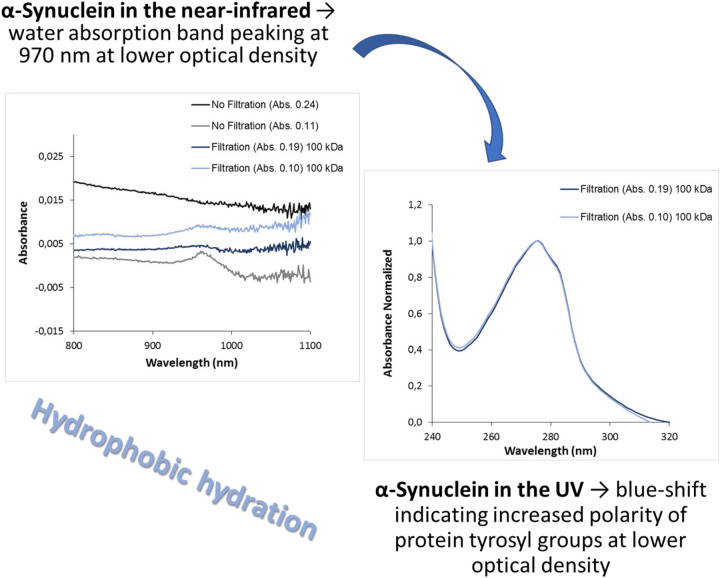
This is a visual representation of the abstract.

## Introduction

Early-formed large α-synuclein (α-syn) protein aggregates have been detected in the protein solutions after an incubation of less than 5  min or in the system lag-phase.^1–4^ Additionally, these early-formed protein aggregates are likely to develop hydrophobic residue segments and become solvent-exposed. In this study, near-infrared (NIR) light was utilized here within the wavelength range of 800 to 1100  nm to gather information about the early stages of α-syn amyloid protein aggregation. The low absorption coefficient in the NIR range enables a high penetration depth, allowing for the analysis of thicker samples (longer path length), and it facilitates the examination of strongly absorbing and even highly scattering samples, such as turbid liquids or solids, in both transmittance and reflectance modes without the need for sample pretreatment. The aggregation of α-syn amyloid protein is involved in the progression of Parkinson’s disease. In the recorded analogous NIR spectra of water and aqueous buffer solutions, a distinct band with absorption maxima reaching 970  nm was noted.^5^ Unexpectedly, a weakened absorption band with peaks around 970  nm was observed in the NIR of the *N*_α_-acetyl-L-tyrosinamide (NAYA) compound and the α-syn protein solutions. The NAYA compound serves as a suitable model for α-syn as it resembles the peptide bonds of proteins and contains a mainly hydrophobic tyrosyl group that is similar to the four tyrosyl groups found in the α-syn molecular structure (there are no tryptophan residues in the protein). Consequently, I used in this research, the hydrophobic hydration concept, which was primarily observed to occur in the analyzed NAYA parent compound solutions. During the examination of the NIR spectra of α-syn protein solutions at the onset of the protein incubation (5  min), hydrophobic hydration was detected as well, although it manifested differently in terms of protein concentration and other properties compared to those observed for the NAYA parent compound.^6^ In this regard, small hydrophobic solutes, such as the NAYA parent compound, can be accommodated into the open hydrogen-bonded network of liquid water without significant perturbations. However, as the solute size increases, such as in early-formed α-syn protein aggregates, water de-wets on the surface of the solute.^6^ Near sufficiently large solutes, the solute–water interface resembles that between vapor and liquid water, and, therefore, requires interfacial thermodynamics for its description.^6^ Correspondingly, the thermodynamics of hydration changes gradually from entropic for small solutes to enthalpic for large solutes.^6^ Additionally, the small-solute hydration is governed by microscopic density fluctuations, whereas large solute hydration is described by the thermodynamics of interface formation.^6^ These two fundamentally different mechanisms will display different dependencies on thermodynamic conditions, thereby providing a means to manipulate the crossover length scale and the associated driving forces. Overall, details concerning the aggregation process at early stages of the α-syn protein were obtained, including the assessment of the size parameters of the α-syn particles in solution by using absorption spectroscopy and the concept of light transport in the protein samples. It appears that this protein system initially evolves to stasis at low protein concentrations, and only at the physiological concentration of α-syn, such as that found in neuronal synapses,^7^ approximately 50  µM, does the protein system become more dynamic and prone to aggregation.

## Experimental

### Materials and Methods

All chemicals, including NAYA, were of analytical grade and obtained from Sigma-Aldrich. Ultrapure water was generated using a Milli-Q Plus Water Purification System (18.2 MΩ·cm).

### *α*-Syn Expression and Purification

The pT7-7 plasmid containing the human α-syn sequence (kindly provided by Professor Doctor T. Outeiro, IMM, University of Lisbon) was used to overexpress α-syn in *Escherichia coli* BL21(DE3) bacteria. Syn was purified as previously described.^1,2,8^

### Sample Preparation

For sample preparation, 600 µL of solution (prepared with high-purity Milli-Q water) was carefully transferred to a quartz cuvette (5  mm path length), and the experiments were performed at room temperature. Water, buffer solutions, NAYA compound solutions, and α-syn protein solutions were obtained and/or prepared on the same day as the spectroscopic measurements. The pH of the protein solution was adjusted prior to measurement. In the case of the protein solutions, the time elapsed between centrifugation (see below) and sample preparation was noted to be 5 minutes. Therefore, protein solutions were aged for 5 minutes before the spectroscopic measurement.

The α-syn stock solutions used in the spectroscopic measurements were filtered by centrifugation with a 100  kDa molecular weight cut-off (MWCO) membrane centrifuge filter (Amicon; the membrane was made of regenerated cellulose), centrifugation conditions: 5000  rpm, or 3214  *g*, relative centrifugal force (RCF), at 4 °C for 3 minutes (1 mL protein stock sample), fixed-angle rotor, Eppendorf Centrifuge 5810). A simple protocol based on filtration through a 100  kDa MWCO membrane has been reported to provide an efficient method for producing an aggregate-free α-syn preparation and for the rapid removal or isolation of α-syn oligomers (> α-syn dimers).^9^

### Spectroscopic Measurements

Ultraviolet (UV) absorption and NIR spectra were recorded using an Agilent Cary 8454 UV–visible (UV–Vis) spectrophotometer with 1.0  nm resolution. For absorption, 5  mm path length quartz fused cuvettes were used. The obtained spectra were corrected with the appropriated references.

## Results and Discussion

### Interpretation of NIR Spectra in the Wavelength Region from 800 to 1100  nm for Liquid Samples

**Figure 1.**
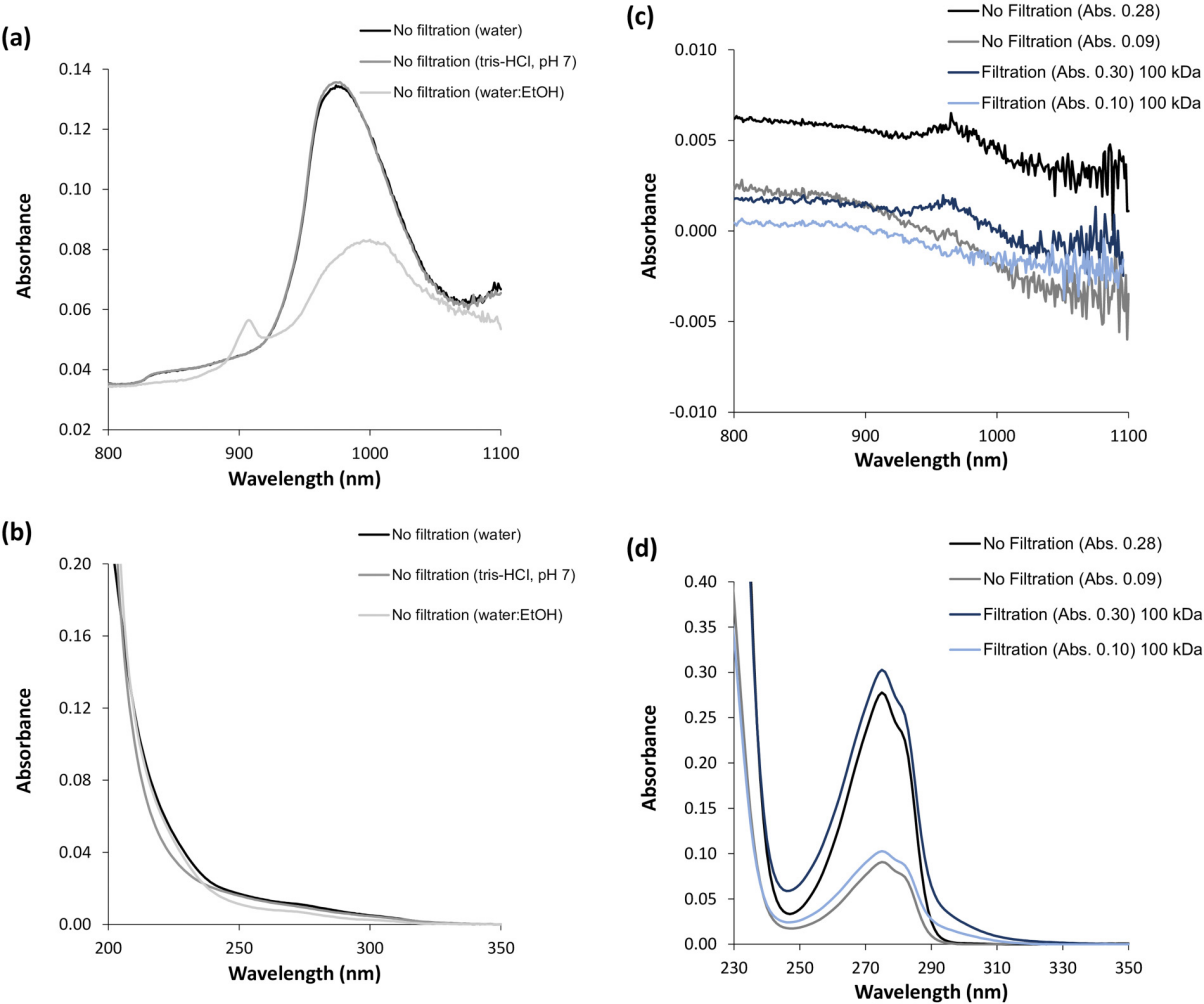

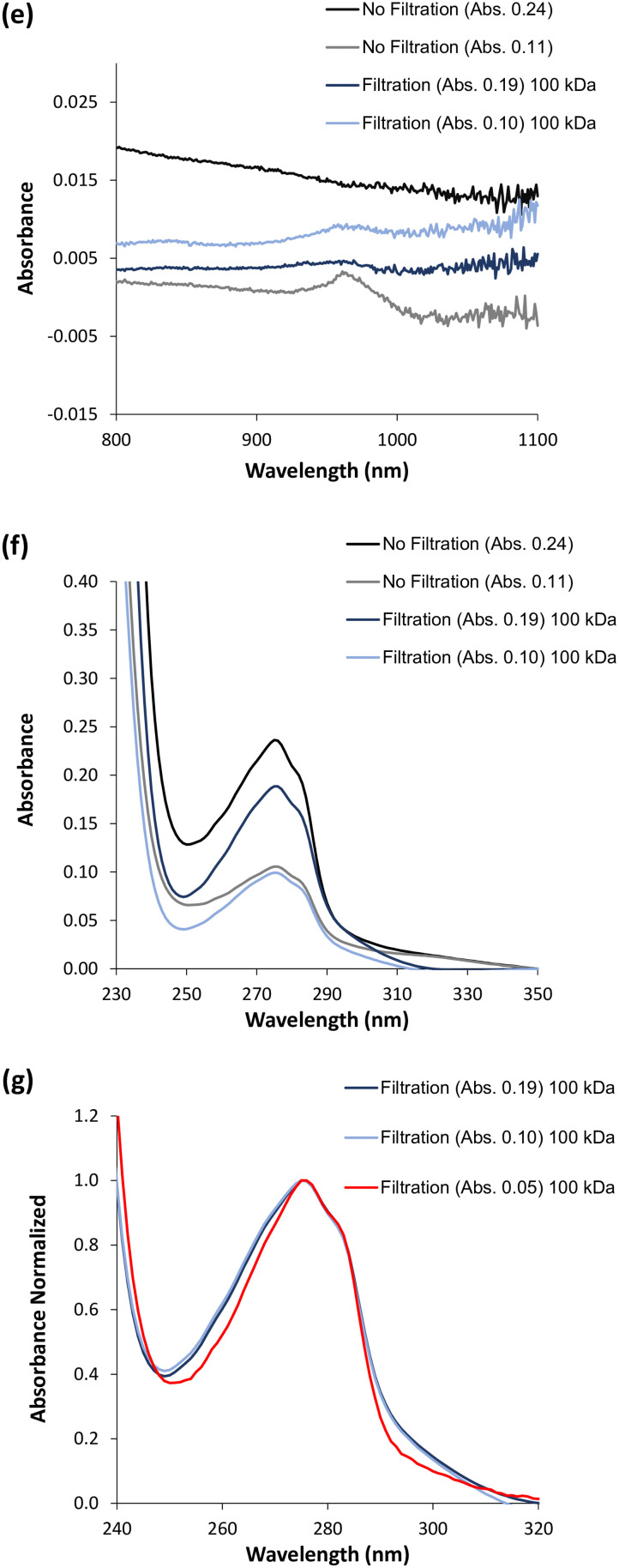
Near-infrared (wavelength region from 800 to 1100  nm) and UV spectra of liquid samples. (a) Near-infrared spectra of water, of an aqueous buffer solution (10  mM tris-HCl, pH 7.0), and of a mixture of water:ethanol (30:70%) (v/v). (b) UV absorption spectra of the same liquid samples referenced in (a). (c) Near-infrared spectra obtained for the NAYA parent compound using different compound concentrations and by filtering or not filtering the solutions. (d) UV absorption spectra of the same solutions referenced in (c). (e) Near-infrared spectra obtained for α-syn protein solutions (5  min of incubation) using different protein concentrations and by filtering or not filtering the solutions. (f) UV absorption spectra of the same solutions referenced in (e). (g) Normalized UV absorption spectra for different α-syn protein concentrations while subjecting the protein solutions to centrifugation. The filtration of NAYA compound and α-syn protein solutions was performed by centrifugation using 100  kDa MWCO (molecular weight cut-off) filters with membranes made from regenerated cellulose. Filtration conditions by centrifugation are as described in Material and Methods section.

There are five prominent water absorption bands in the NIR spectrum, with absorption maxima at 760, 970, 1190, 1450, and 1940 nm.^5^ In this study, the wavelength region from 800 to 1100  nm was monitored, encompassing the expected water absorption band at 970  nm. In this regard, it was decided to focus on the water absorption band at 970  nm to elucidate the phenomena pertaining to the hydrophilic environment sensed by the NAYA parent compound and the α-syn amyloid protein. Furthermore, in Figure 1a is shown the recorded NIR spectra (in the wavelength region: 800–1100  nm) of water, of an aqueous buffer solution (10  mM tris-HCl, pH 7.0), and of a mixture of water:ethanol, 30:70% (v/v). It is known from the literature that different types of functional groups, such as C−H, N−H, and O−H, with various energy levels can absorb near-infrared light according to their unique vibrational frequencies.^10^ Thus, in Figure 1a, it can be observed the characteristic absorption band of water peaks at 970  nm in the NIR, and this spectrum is similar to that of the tested aqueous buffer solution (10 mM tris-HCl, pH 7.0). I also tested a solution containing the mixture of water:ethanol, 30:70% (v/v), and indeed, the characteristic absorption band of ethanol peaks at 910  nm, accompanied by a decrease in the intensity of the absorption band of water peaking at 970  nm (Figure 1a). In Figure 1b, the UV absorption spectra of the same samples depicted in Figure 1a are presented, and it is clear that these samples only absorb significantly for wavelengths below 250  nm, as to be expected. Moreover, in Figure 1c, the near-infrared spectra of the NAYA parent compound in water, both with and without filtration under centrifugation conditions, are shown. In fact, the near-infrared spectra of the NAYA parent compound is independent whether or not the filtration process is executed under centrifugation conditions (Figure 1c). Additionally, a small absorption band appears in the near-infrared spectra, peaking at approximately 970  nm, particularly when using higher concentrations of the NAYA parent compound. Previously, I mentioned that water absorbs in the near-infrared, revealing a band peaking at 970  nm. Since the NAYA parent compound molecule contains a tyrosyl group in its molecular structure, which is mainly hydrophobic, increasing the concentration of NAYA likely leads to an increase in the hydrophobic content of the compound solution. Therefore, it is possible that in more concentrated NAYA solutions, water molecules can organize in the vicinity of NAYA compound molecules, and this phenomenon may actually represent a spectral signature of such compound solutions. Furthermore, in Figure 1d, the UV spectra of the same samples used in Figure 1c are presented, and in these UV spectra, it can be observed that when the NAYA compound solutions are filtered by centrifugation, in particular, there is an evident tail resulting from the regenerated cellulose absorption band, with maximum intensity peaking at approximately 300  nm.^11^ Moreover, in Figure 1e, the NIR spectra obtained for the α-syn protein under different protein concentrations, filtered or unfiltered by centrifugation, are shown (5  min of incubation). Similarly, to Figure 1c, the recorded near-infrared spectra of α-syn appear independent of the filtration; in fact, a small absorption band peaking at approximately 970  nm appears in the spectra for lower protein concentrations (Figure 1e). Previously, in Figure 1c, it was mentioned that a similarly small absorption band peaking at approximately 970  nm was identified in the near-infrared spectra of the NAYA compound, but at a higher concentration of this compound. In the case of α-syn, the aforementioned small absorption band peaking at approximately 970  nm in the near-infrared spectra, at lower protein concentrations, can be attributed to the specific ordering of water molecules in the vicinity of hydrophobic residues of early-formed protein aggregates. In Figure 1f, the recorded UV absorption spectra for the same protein solutions depicted in Figure 1e are presented. It can be observed in Figure 1f that without filtration by centrifugation of the α-syn solutions, there is a pronounced tail, as expected, accounting for the presence of impurities and/or protein aggregates developed during the protein purification steps. With filtration by centrifugation of the α-syn solutions, the referred tail is significantly reduced (Figure 1f), as anticipated. Also, from Figure 1g, a quasi-imperceptible blueshift (< 1 nm) in the filtered α-syn protein solutions is noticeable when there is a decrease in the protein concentration (*A*_275 nm_ = 0.19 to *A*_275 nm_ = 0.10), indicating a small increase in the effective polarity around the protein’s tyrosyl groups.^8^ This result is consistent with the previously mentioned interpretation of the near-infrared spectra of α-syn, where an increase in ordering of water molecules in the vicinity of the hydrophobic residues in the early-formed protein aggregates was determined for the lower protein concentrations investigated. However, when further decreasing the α-syn protein concentration (*A*_275 nm_ = 0.05), a different scenario emerges, in which there is a noticeable redshift in the filtered protein solution. This accounts for a decrease in the effective polarity in the vicinity of the α-syn protein’s tyrosyl groups, indicating that these tyrosyl groups sense a more hydrophobic environment. This behavior, in fact, contradicts the expected tendency of the hydrophilic environment sensed by the early-formed protein aggregates when decreasing the protein concentration. This effect can be related to less solvent-exposed hydrophobic residues while decreasing the protein concentration, leading to the canceling of the hydrophobic hydration in early-formed protein aggregates.

### Hydrophobic Hydration of Early-Formed α-Syn Protein Aggregates in the NIR

**Figure 2.**
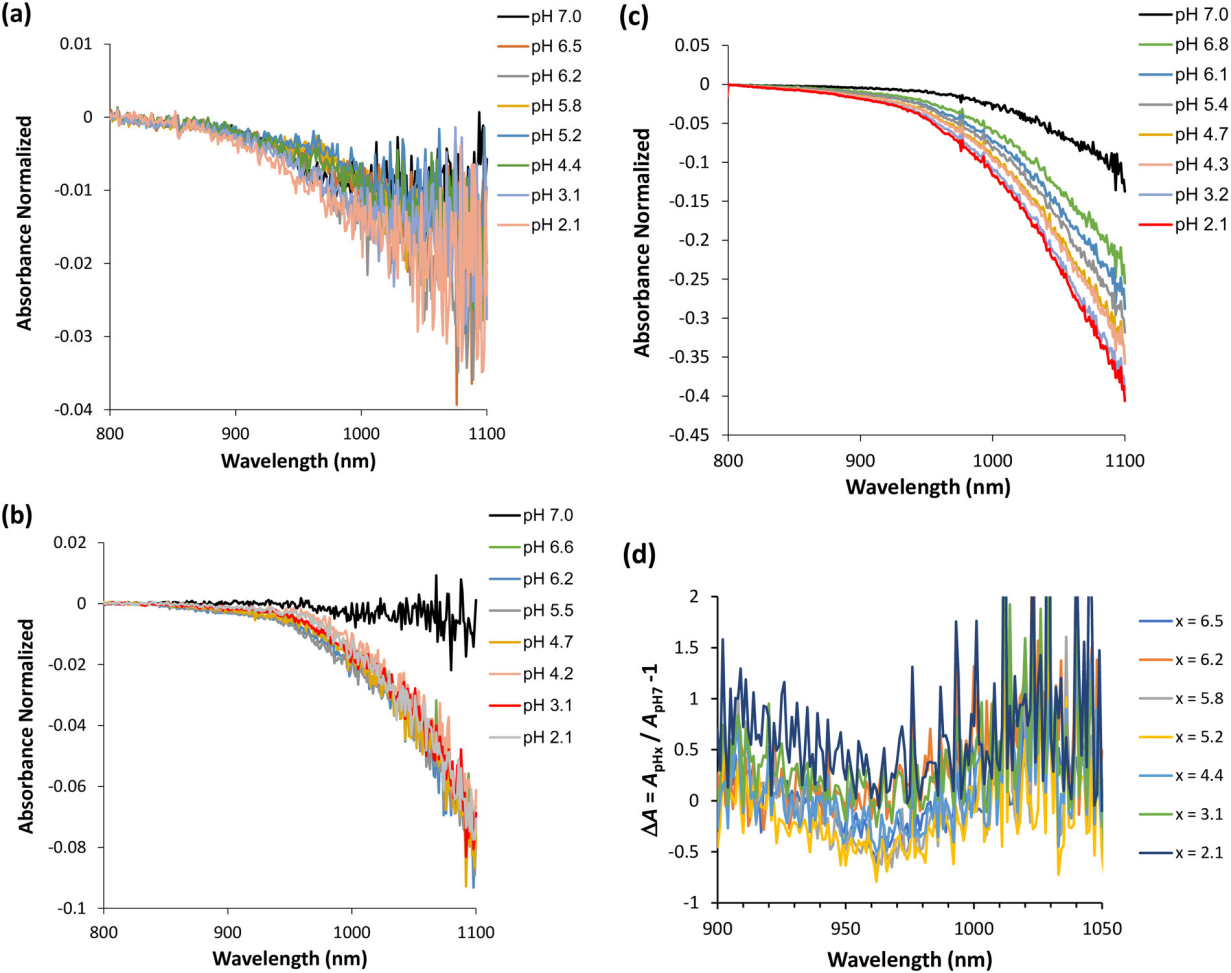

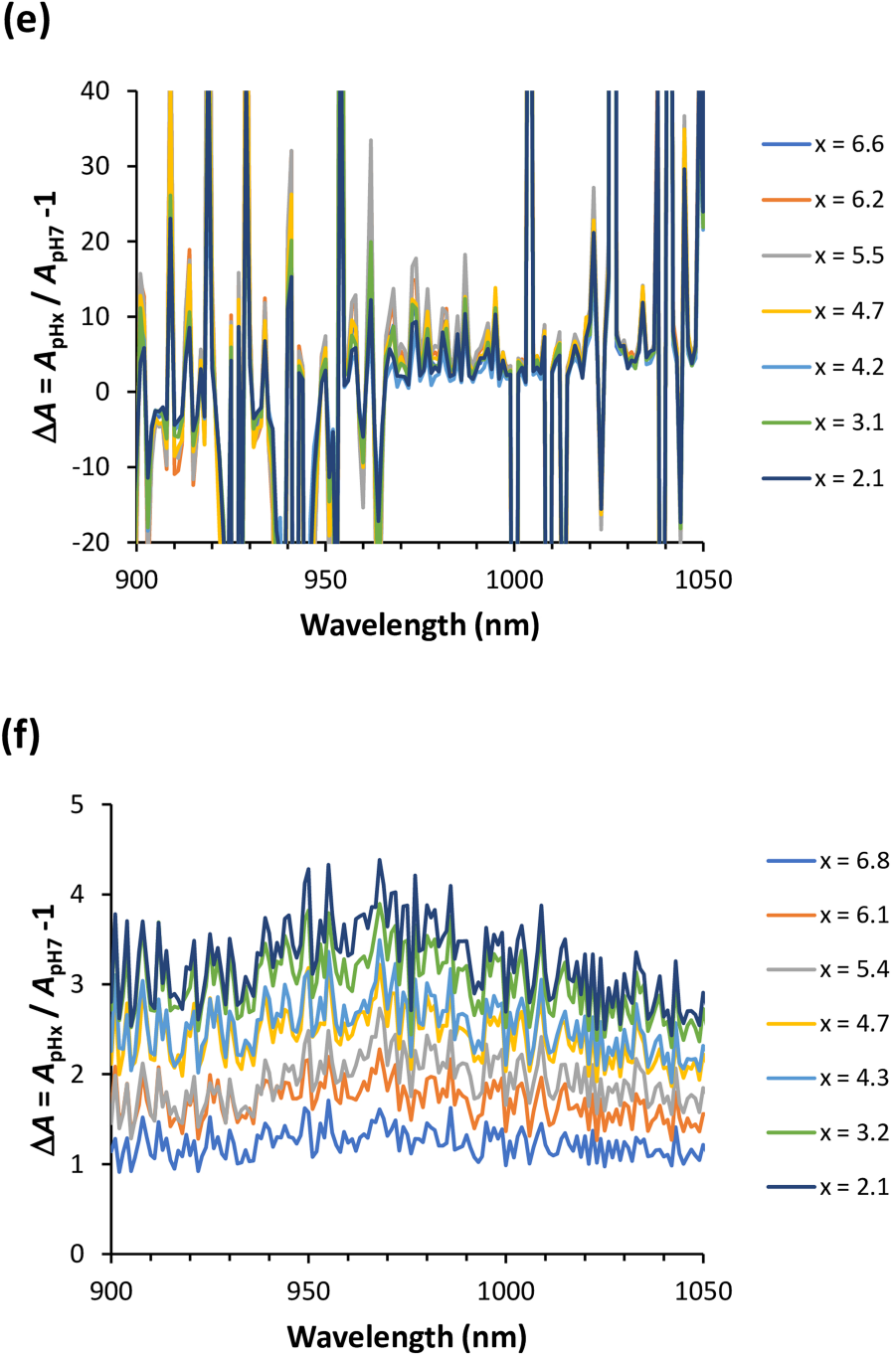
Near-infrared spectra were collected in the wavelength region from 800 to 1100  nm of the α-syn protein while varying the pH of the protein solution from 7.0 to 2.1 (5  min of incubation). (a) *A*_275 nm_ = 0.15; ε = 5974 M^−1^ cm^−1^; protein concentration of 50.2  µM. (b) *A*_275 nm_ = 0.10; ε = 5974 M^−1^ cm^−1^; protein concentration of 33.5  µM. (c) *A*_275 nm_ = 0.05; ε = 5974 M^−1^ cm^−1^; protein concentration of 16.7  µM. (d–f) absorbance variation, Δ*A* for (a–c), respectively.

Next, it was decided to study the effect of varying the pH of the protein solution at different protein concentrations by recording the near-infrared spectra. Indeed, an increase in the effective polarity in the vicinity of α-syn tyrosyl groups was previously noted; this occurred at a lower optical density (OD) than that observed for the NAYA parent compound (Figures 1c and 1e). This indicates that it is essential to consider not only the tyrosyl groups present in the α-syn molecular structure but also the existence of interacting hydrophobic segments (containing tyrosyl groups) that develop during the course of the protein aggregation process. It is also important to mention that the tyrosyl group possesses an attached hydroxyl, which is acidic, and can establish hydrogen-bonds with water molecules. In this context, the topic of hydrophobic hydration has been addressed in the literature,^12–14^ noting that the presence of small hydrophobic solutes in water (such as nonpolar gases) primarily results from changes in the clustering in the surrounding water and the consequential difference in energy between these water molecules and the bulk water, rather than from water-solute interactions.^15^ Hydrophobic hydration leads to a reduction in density and an increase in heat capacity.^16,17^ The expanded network causes the density to decrease, while the ordered bonds must bend when the temperature increases, thereby affecting the heat capacity. Thus, hydrophobic hydration behaves oppositely to polar hydration, as polar hydration increases the density and decreases heat capacity due to their associated disorganized hydrogen bonds being already being bent or broken. Within this hydrophobic hydration of tyrosyl groups, one can infer that even a slight increase in the proton concentration, such as when lowering the pH of the Syn solution (Figure 2), can disturb the aforementioned ordering of water molecules around the tyrosyl groups, which is also dependent on the density of early-formed protein aggregates. Furthermore, in Figures 2a to 2c (Figure S1, Supplemental Material), the normalized absorbance values as a function of the wavelength range corresponding to the utilized NIR light are presented. The negative values of normalized absorbance indicate for the occurrence of scattering in the α-syn protein solutions (5  min of incubation). As the concentration of the protein solutions decreases, from Figures 2a to 2c, the normalized absorbance values become less scattered and indicate for increased scattering in those solutions, i.e., when the protein concentration decreases. In addition, I further determined the variation of the normalized absorbance values, in terms of Δ*A* (Figures 2d to 2f). In Figure 2d, which corresponds to the highest protein concentration investigated (*A*_275 nm_ = 0.15; 50.2  µM) (Figure 2a), only for protein solutions with pH ≥ 4.4 does Δ*A* present slightly negative values around 970  nm. This may indicate that the early-formed α-syn protein aggregates still sense the hydrophobic hydration of their hydrophobic residues and protein aggregation is expected to continue. In Figure 2e, which corresponds to the intermediate protein concentration investigated (*A*_275 nm_ = 0.10; 33.5 µM) (Figure 2b), the calculated Δ*A* values are positive around 970  nm for the pH range studied. Therefore, it can be inferred that hydrophobic hydration reports on consecutive events of intramolecular interactions in the early-formed protein aggregates, and in this case, aggregation may not proceed; hydrophobic hydration can be canceled. In Figure 2f, which corresponds to the lowest protein concentration investigated, *A*_275 nm_ = 0.05; 16.7  µM (Figure 2c), the determined Δ*A* values are positive around 970  nm, particularly for pH ≤ 3.2. Thus, the occurrence of hydrophobic hydration entails again on consecutive steps of intramolecular interactions in the early-formed α-syn protein aggregates as similarly discussed in Figure 2e. Additionally, it should be borne in mind that for pH ≤ 3.2, the α-syn protein experiences the intramolecular hydrophobic collapse, wherein the neutralized C-terminal region collapses with the NAC region,^18,19^ which is evidence for intramolecular interactions in the early-formed protein aggregates. From this discussion, one can deduce that intermolecular and intramolecular interactions in the early-formed α-syn protein aggregates are mainly favored at the highest concentration investigated (50.2  µM) and at the lower concentrations investigated, 33.5  µM and 16.7  µM, respectively. However, for the latter two protein concentrations, the hydrophobic hydration signature encountered in this system can be disappear due to the consecutive steps of intramolecular interactions in the early-formed α-syn protein aggregates while they achieve further stability.

### Interpretation of the Reduced Scattering of the α-Syn Protein Solutions and Determination of Parameters Related to the Composition of Scattered Protein Species

Next, I applied the concept of light transport of in the α-syn protein samples, which is governed by irradiation and the geometry of α-syn protein species, as well as by the optical parameters of the protein samples, including their refractive index *n_m_*, their scattering asymmetry (or anisotropy) factor *g*, their scattering coefficient µ*_s_*, and their absorption coefficient µ*_a_*.^20^ The optical scattering parameters, specifically the *g*-factor and the scattering coefficient, are defined as the average cosine of the photon scattering angle and the probability of photon scattering per unit path length, respectively.^20^ The effective scattering is often quantified by weighting the scattering coefficient with an angular scattering factor to yield the reduced scattering coefficient, µ*_s_*’ = µ*_s_* (1 − *g*). The optical absorption parameter, namely the absorption coefficient, is defined as the photon absorption per unit path length. Additionally, further information could be obtained from the transport scattering spectra.^20^ For a homogeneous sphere of radius *r*, Mie theory predicts the wavelength dependence of the scattering and the relation between scattering and sphere size.^21^ Under the hypothesis that the scattering centers are homogeneous spheres behaving individually, the relationship between the reduced scattering coefficient, µ*_s_*’, and wavelength (λ) can be empirically described as follows:^22^

(1) $$\mu_{S}^{\prime }=ax^{b}$$

where the size parameter *x* is defined as *x* = 2π*rn_m_*λ^−1^, with the refractive index of the medium *n_m_* chosen to be 1.35, and *a* and *b* are free parameters.^23^ In particular, *a* is proportional to the density of the scattering centers, while *b* depends on their size. Then, a third-order polynomial fit of *b* as a function of *r* can empirically yield the radius of the sphere.^20^ Similarly, the relation between *r* and the scattering anisotropy factor *g* can be empirically described using a third-order polynomial function.^20^ Thus, the evaluated values of *r* allow for an estimate of the *g* factor averaged over the 600–1050  nm range.^20^ Additionally, Eq. 1 can also be used in the form:

(2) $$\mu_{S}^{\prime }(\lambda )=k\lambda^{n}$$

where, in fact, *k* is a variable and *n* = −*b*. Eq. 2 can be rearranged as

(3) $$\ln\;[\mu_{S}^{\prime }(\lambda )]=\ln\;(k)+n\;\ln(\lambda )$$

Furthermore, Eq. 2 was applied to the recorded near-infrared spectra displayed in Figures 2a to 2c, but for the shorter wavelength region from 1050 to 1100  nm. This was due to the following aspects: (i) In the referenced shorter wavelength region, there is minor contribution of the reduction of the scattering intensity with the increase of the wavelength, and (ii) in the stated shorter wavelength region, there is actually an enhanced variation of the recorded absorbance values. Moreover, Figures S2 to S4 (Supplemental Material) show the near-infrared spectra in the wavelength region from 1050 to 1100  nm as presented in Figures 2a to 2c, respectively, and the corresponding fits through the application of Eq. 2. Additionally, in Tables S1 to S3 (Supplemental Material), the α-syn protein size parameters, determined through the application of Eq. 2 to the collected near-infrared spectra in Figures 2a to 2c, respectively, are summarized, particularly in the wavelength region from 1050 to 1100  nm. In Tables S4 and S5 (Supplemental Material), the values obtained for the particle radius (nm) of the α-syn aggregated species, i.e., for the highest and for the intermediate α-syn protein concentrations investigated (Figures 2a and 2b), through the application of the third-order polynomial fits of *n* as a function of the radius *r* (Eqs. 4 and 5), are indicated, yielding, in turn, the particle radius of the homogeneous sphere empirically.^20^ Therefore, the third-order polynomial function for the small and intermediate-sized particle region is:

(4) $$\begin{array}{r} n=-1109.5r^{3}+341.67r^{2}-9.3696r-3.9359\\ \;(r<0.23\;\mu m) \end{array}$$

and the other third-order polynomial function for the larger particle region

(5) $$\begin{array}{r} n=23.909r^{3}-37.218r^{2}+19.534r-3.965\\ \;(0.23\leq r<0.60\;\mu m) \end{array}$$

**Figure 3.**
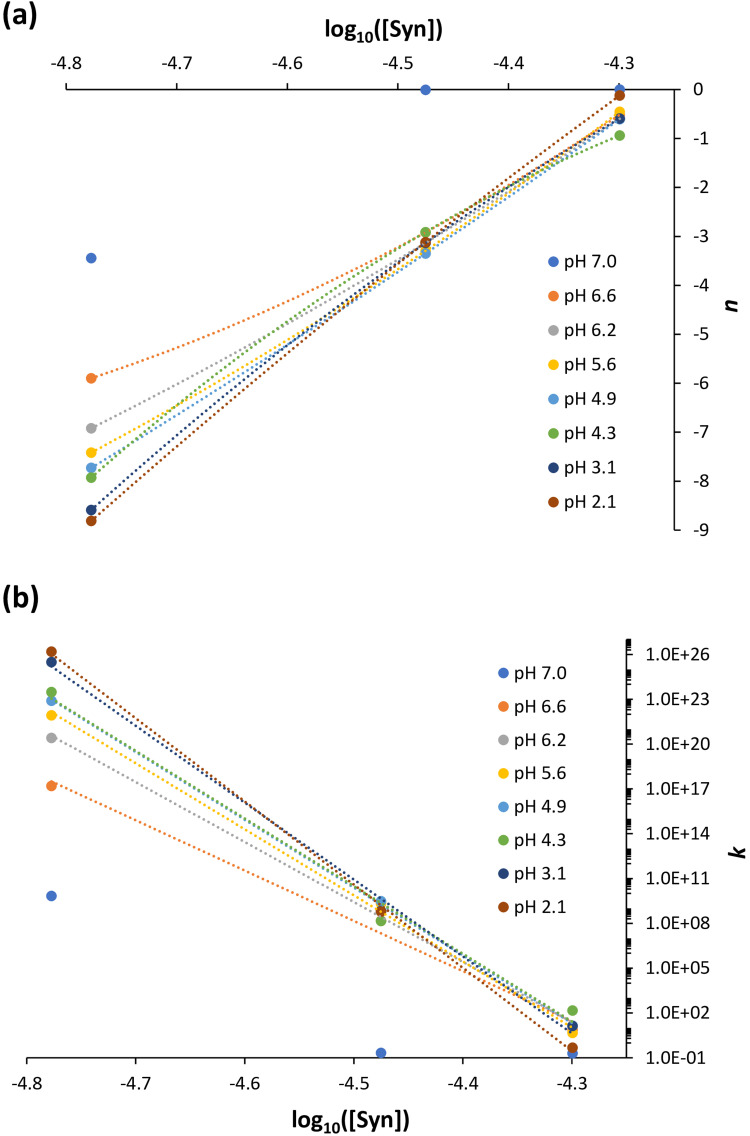
α-Syn protein size parameters, *n* (dependent on the size of the particle), and *k* (proportional to the density of the scattering centers) calculated using Eq. 2 as a function of the protein concentration. (a) *n* generally decreases almost linearly with the decrease in protein concentration (the particle size increases with increasing protein concentration). (b) *k* generally increases exponentially with the decrease in protein concentration (the scattering centers density decreases with increasing protein concentration). Polynomial and exponential fits were applied in (a) and (b), respectively. Protein solution pH: 7.0 ± 0.0; 6.6 ± 0.2; 6.2 ± 0.1; 5.6 ± 0.2; 4.9 ± 0.3; 4.3 ± 0.1; 3.1 ± 0.1; 2.1 ± 0.0.

Therefore, in Figure 3a it can be observed that the calculated *n* parameter values, in which *n* is dependent on the particle size, vary almost linearly with the α-syn protein concentration for pH < 7. In particular, it can be inferred that the size of the protein particles decreases almost linearly with the decrease of the protein concentration. Conversely, in Figure 3b, the calculated *k* parameter values, in which *k* is proportional to the density of the scattering centers, increase exponentially with the decrease of the protein concentration for pH < 7. Given that I previously mentioned that α-syn protein aggregation is particularly favored at the highest concentration investigated (50.2  µM), it can be deduced that less dense particles are more prone to aggregation than denser and more organized particles. In this scenario, one can assume that these less dense particles are more dynamic, facilitating the establishment of intermolecular interactions, whereas the aforementioned denser particles, at the intermediate (33.5  µM) and lowest protein concentrations (16.7  µM) studied, primarily involve the occurrence of intramolecular interactions. Thus, at these latter two protein concentrations, the system is more stable and likely to be more homogeneous.

**Figure 4.**
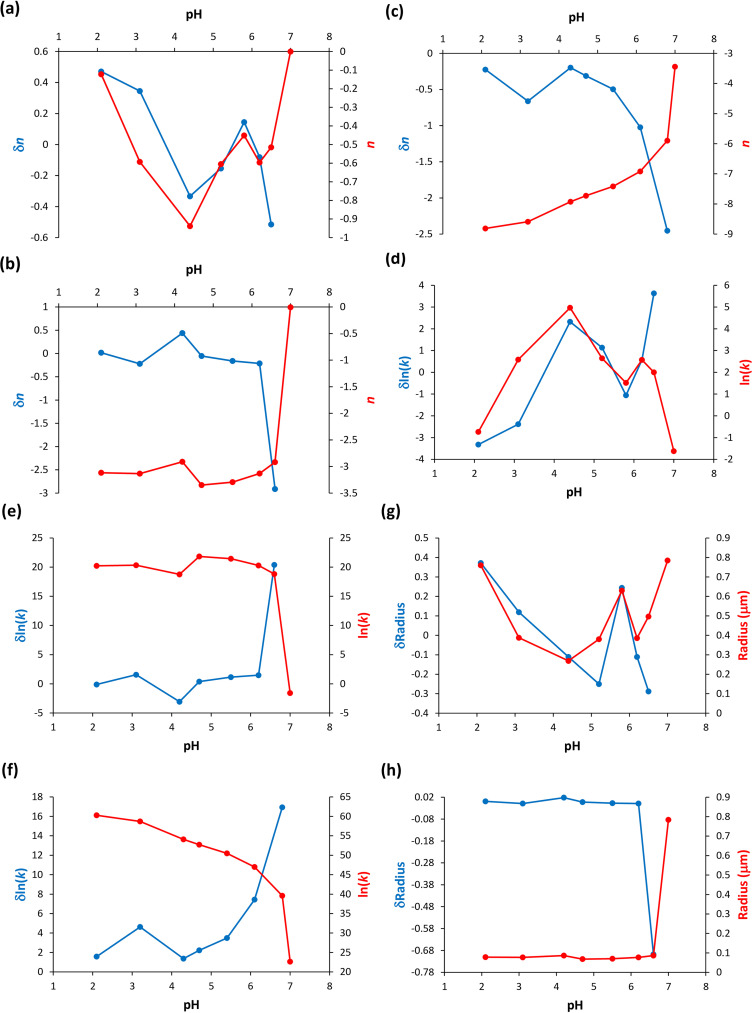
α-Syn protein particle size parameters calculated using Eq. 2 as a function of the pH of the protein solution. These particle size parameters are indicated in Tables S1, S2, and S3 (Supplemental Material). (a) Values of *n* and of their variation, δ*n*, for the highest protein concentration investigated 50.2  µM. (b) Values of *n* and of their variation, δ*n*, for the intermediate protein concentration investigated (33.5 µM). (c) Values of *n* and of their variation, δ*n*, for the lowest protein concentration investigated (16.7  µM). (d) Values of *k* and of their variation, δln(*k*), for the highest protein concentration investigated (50.2  µM). (e) Values of *k* and of their variation, δln(*k*), for the intermediate protein concentration investigated (33.5  µM). (f) Values of *k* and of their variation, δln(*k*), for the lowest protein concentration investigated (16.7  µM). (g) Values of the particle radius (µm) and of their variation, δradius, for the highest protein concentration investigated (50.2  µM), see Table S4 (Supplemental Material). (h) Values of the particle radius (µm) and of their variation, δradius, for the intermediate protein concentration investigated (33.5  µM), see Table S5 (Supplemental Material).

Next, the *n* and *k* parameters determined through the application of Eq. 2 were represented as a function of the protein solution pH, i.e., across the different protein concentrations investigated (Figure 4). In Figure 4a, for the highest protein concentration examined (50.2  µM), the size of the protein particles, in terms of *n*, does not monotonically decrease for pH ≥ 4.4, and this result corroborates the earlier mention that intermolecular interactions in the early-formed protein aggregates always translate hydrophobic hydration (Figure 2d). Additionally, in Figures 4b and 4c, for the intermediate (33.5  µM) and lowest (16.7  µM) protein concentrations examined, the size of the protein particles, as indicated by *n*, decreases monotonically in the pH range studied, which is consistent with the previous discussion that consecutive intramolecular interactions can cancel hydrophobic hydration (Figures 2e and 2f). When determining the variation of *n*, in terms of δ*n*, it is evident that below pH 4 there is a deviation from the monotonically decreasing of the *n* values, which can be interpreted as an increase of the protein particle size (Figures 4b and 4c). This is due to the occurrence of the previously mentioned α-syn protein hydrophobic collapse at pH below 4,^18,19^ encompassing the manifestation of hydrophobic hydration on still existent hydrophobic residues. Moreover, Figures 4d to 4f, highlighting the variation of ln(*k*) values as a function of the protein solution pH, and also in terms of δln(*k*), exhibit a trend opposite to the calculated *n* values, as expected given Figure 3. In Figure 4g, I calculated the radius (µm) of the α-syn particles and their variation, in terms of δradius, for the highest protein concentration investigated (50.2  µM) while using Eq. 5. The obtained trend is similar to the trend observed for the calculated *n* values and of their variation, in terms of δ*n*, depicted in Figure 4a. In Figure 4  h, the calculated the radius (µm) of the α-syn particles and their variation, in terms of δradius, for the intermediate protein concentration investigated (33.5 µM) is illustrated while using Eqs. 4 and 5. The obtained trend is different from the trend observed for the calculated *n* values and of their variation, in terms of δ*n*, depicted in Figure 4b. In this latter situation, it seems that protein particles approach the Mie proportionality at pH 7 and the Rayleigh proportionality at pH < 7.^20^ In other words, the protein solution primarily attains the stability of its molecular species by significantly reducing the particle size, consequently increasing their density, and thereby making the solution more homogeneous. It appears that short- and long-range order effects cooperate to achieve more stable protein particles and make the protein solution more homogeneous instead of promoting protein aggregation. It is however important to note that the α-syn protein hydrophobic collapse does not appear to occur at the highest protein concentration examined (50.2 µM); rather, it occurs at the intermediate (33.5 µM) and lowest (16.7 µM) protein concentrations investigated.

## Conclusion

By examining the initial incubation period (5  min) of the α-syn protein, it was determined that the protein system reaches stasis, particularly at concentrations lower than the one estimated in neuronal synapses (approximately 50 µM),^7^ by enhancing the particle density while simultaneously reducing the particle size. The effects of short- and long-range order appear to cooperate in ensuring the stability of the protein particles in solution. At an approximate protein concentration of 50  µM, the system exhibits increased dynamics and promotes protein aggregation. For the first time, the concept of hydrophobic hydration has been utilized here to elucidate the environment perceived by the interacting hydrophobic residues of the early-formed protein aggregates. Moreover, by merely employing near-infrared light within the wavelength range of 800 to 1100  nm, it becomes feasible to obtain data for this specific protein system at the onset of the protein incubation, thus necessitating the application of absorption spectroscopy and the concept of light transport in the protein samples. This research relies not only on the application of novel concepts to analyze α-syn protein aggregation but also requires conventional technologies and methods to explore the intricate behavior of this amyloid protein aggregation system, which can be found in any research laboratory.

## Supplemental Material

sj-docx-1-asp-10.1177_00037028251367004 - Supplemental material for Hydrophobic Hydration and Light Transport in α-Synuclein Protein Solutions in the Near-Infrared Supplemental material, sj-docx-1-asp-10.1177_00037028251367004 for Hydrophobic Hydration and Light Transport in α-Synuclein Protein Solutions in the Near-Infrared by Marco A. Saraiva in Applied Spectroscopy
