# Supplementary material for: Hydrophobic Hydration and Light Transport in α-Synuclein Protein Solutions in the Near-Infrared
Source: Appl Spectrosc. 2025 Jul 30;79(12):1726–36. doi: 10.1177/00037028251367004 (PMC12634906; doi:10.1177/00037028251367004)
Supplement: sj-docx-1-asp-10.1177_00037028251367004 - Supplemental material for Hydrophobic Hydration and Light Transport in α-Synuclein Protein Solutions in the Near-Infrared [file sj-docx-1-asp-10.1177_00037028251367004.docx]

**Supplemental Material**

**Hydrophobic Hydration and Light Transport in α-Synuclein Protein Solutions in the Near-Infrared**

Marco A. Saraiva^1,2^

^1^Centro de Química Estrutural, Institute of Molecular Sciences, Instituto Superior Técnico, University of Lisbon, 1049-001 Lisbon, Portugal

^2^Instituto de Tecnologia Química e Biológica António Xavier, Universidade Nova de Lisboa, Av. da República, 2780-157 Oeiras, Portugal

* Corresponding author email: marco.saraiva@tecnico.ulisboa.pt

**Figure S1.** UV absorption spectra of the α-syn protein solutions for the various protein concentrations investigated at pH 7.0 (Figures 2a to 2c).


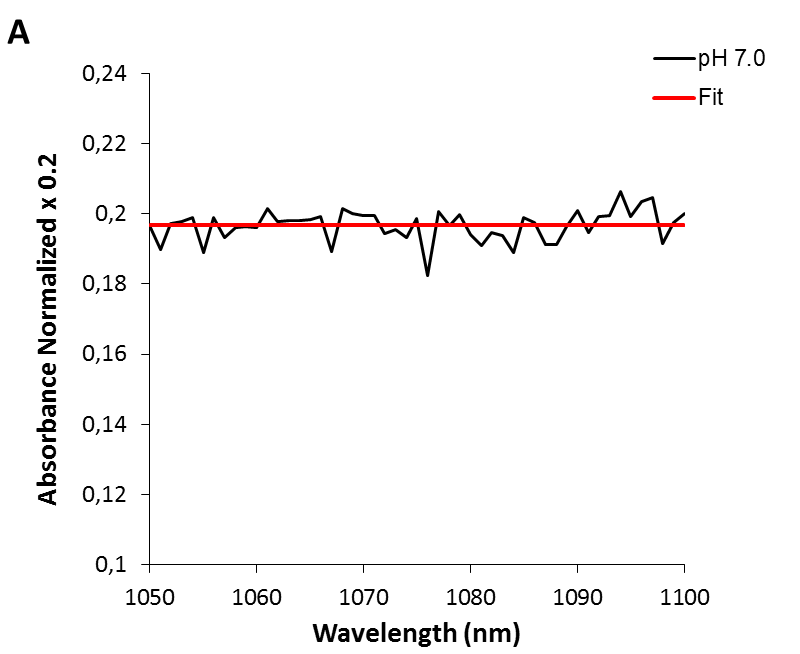

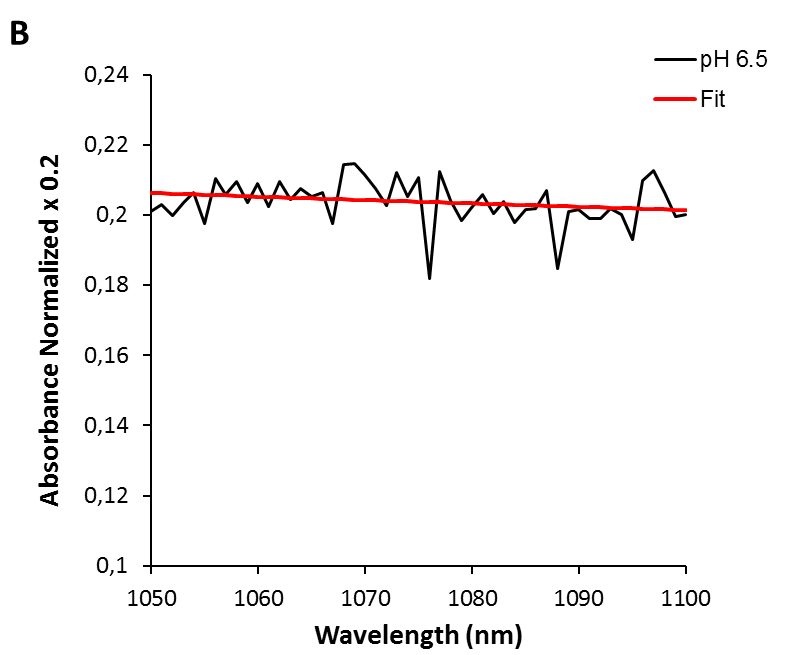


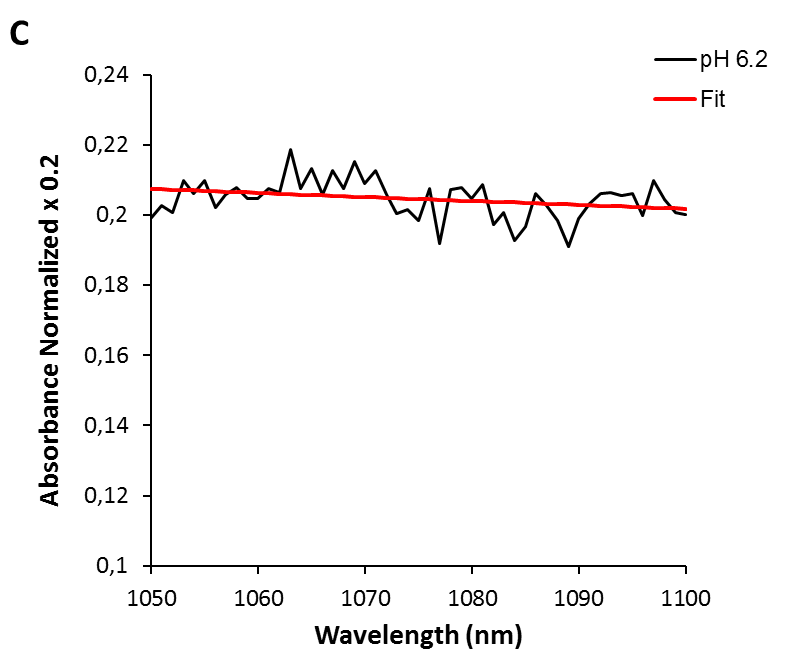

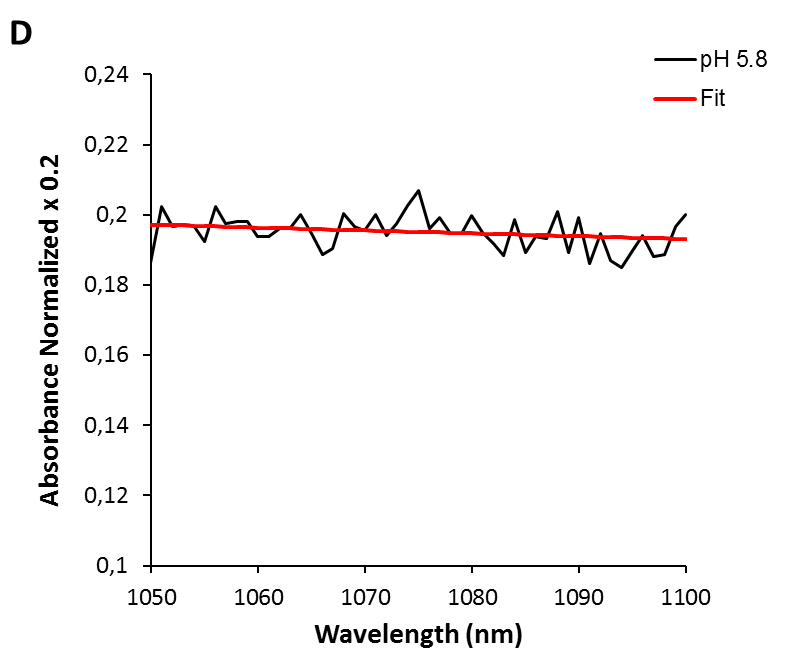


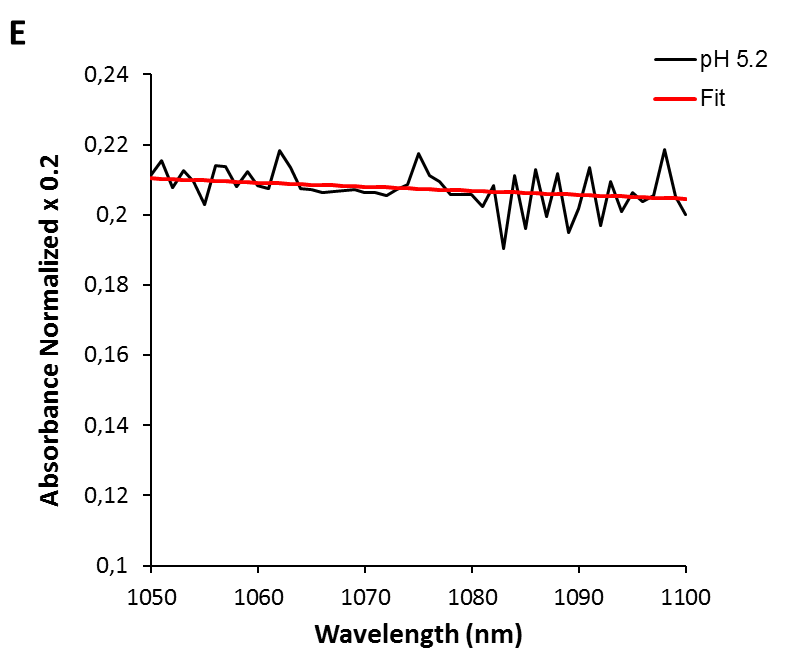

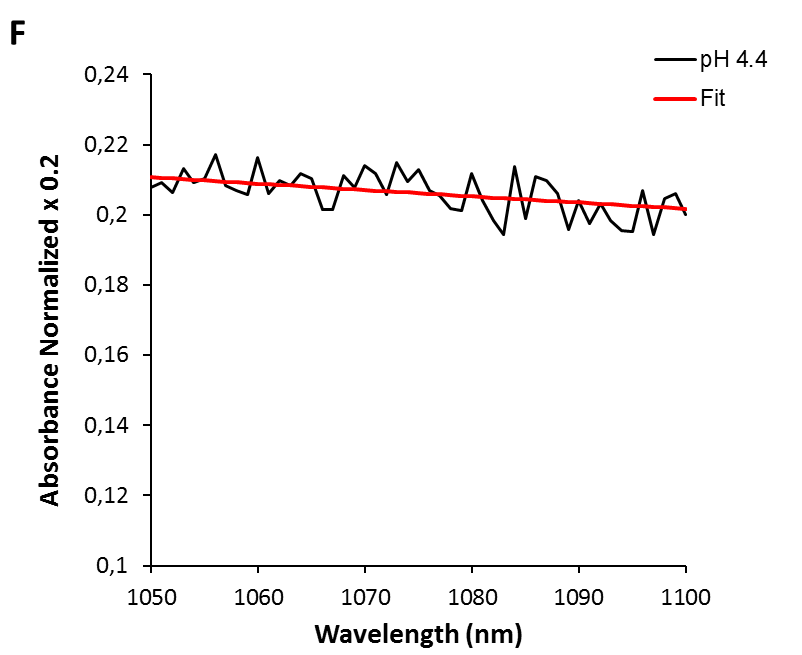


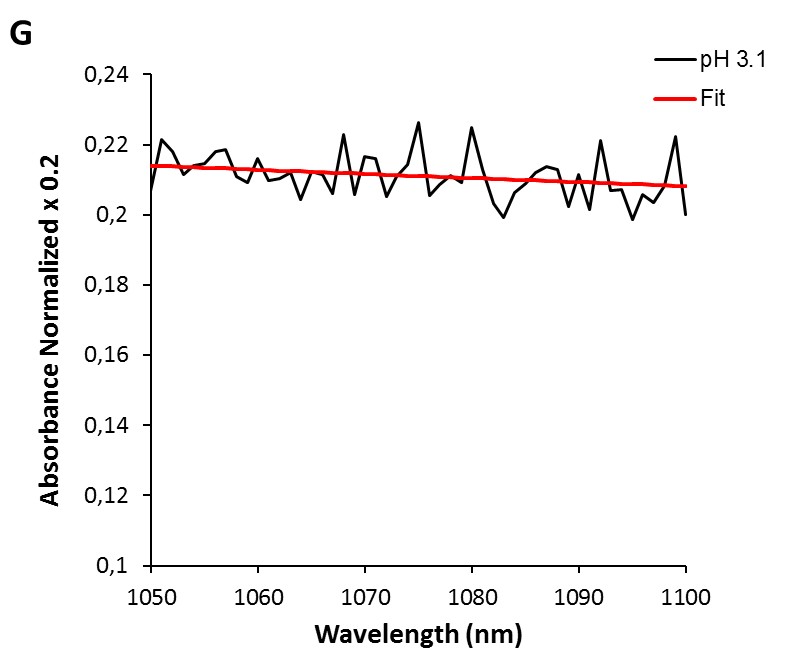

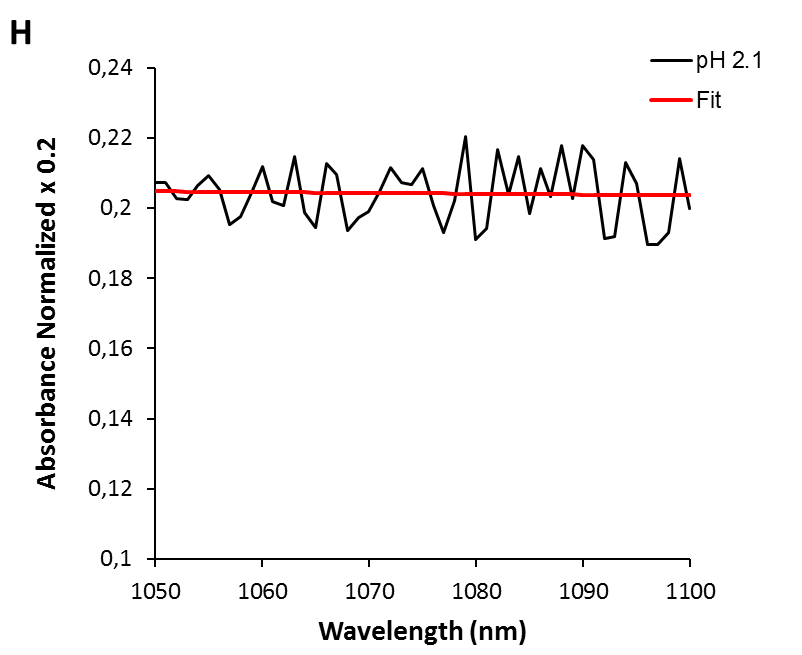


**Figure S2.** Application of Eq. 2 to the NIR spectra in the wavelength region from 1050 to 1100 nm of the Syn protein while varying the pH of the protein solution. (a–h) The normalized NIR spectra displayed in Figure 2a and the corresponding fits, for α-syn protein solutions at pH 7.0 to 2.1.


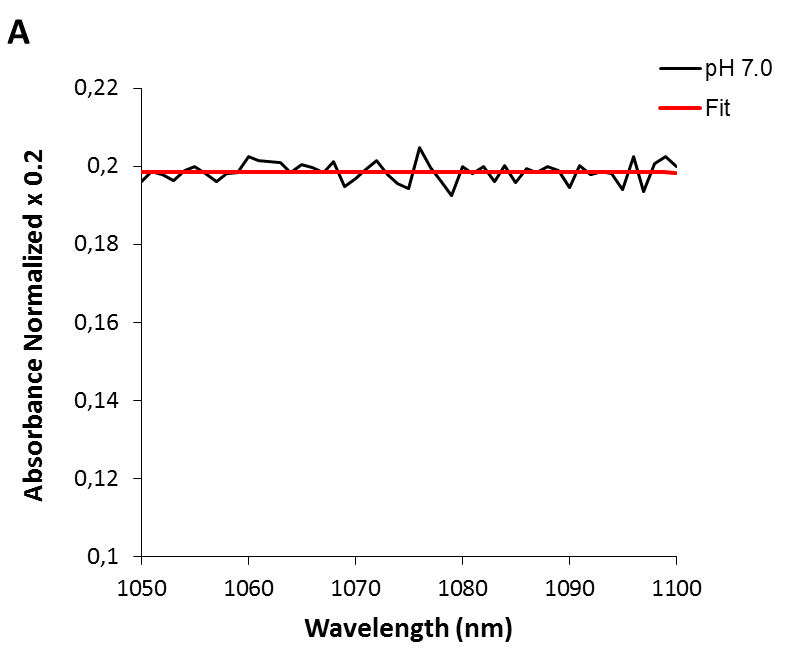

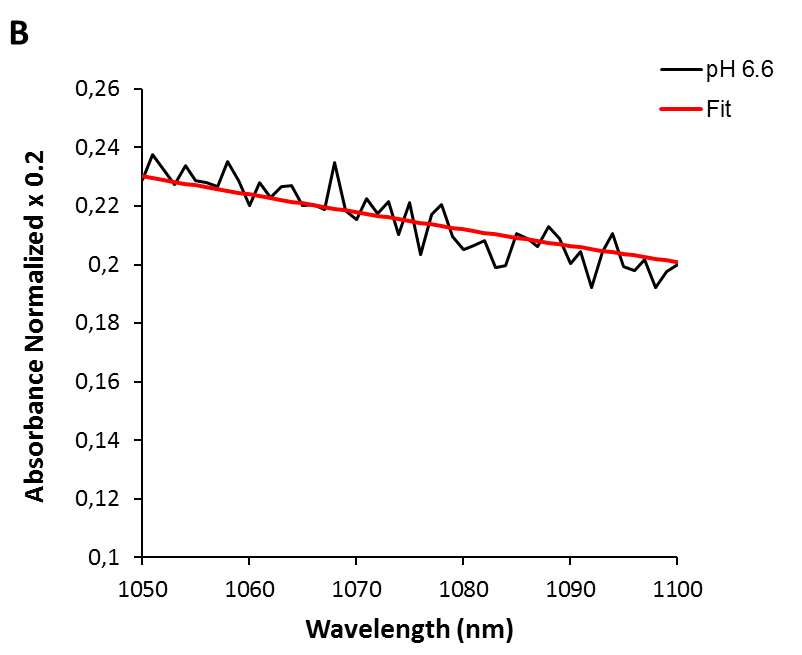


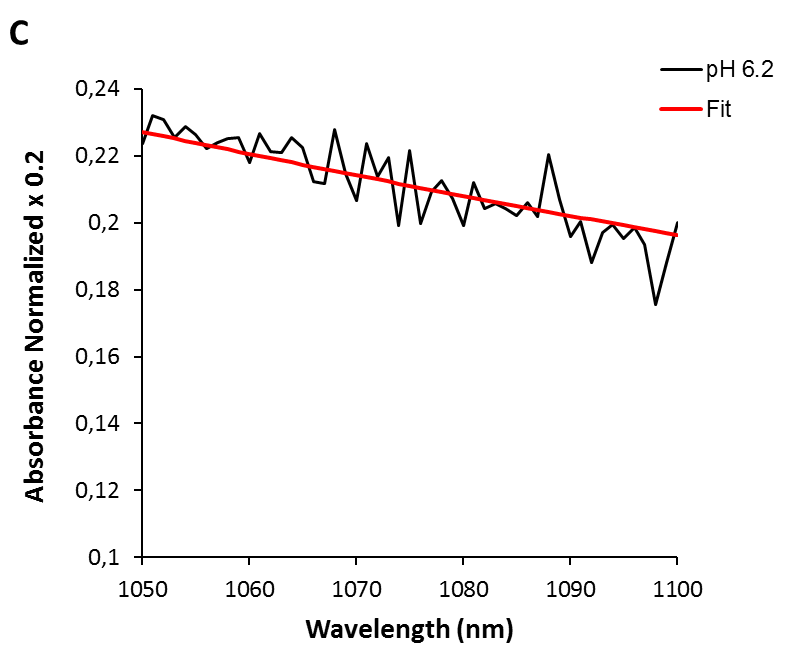

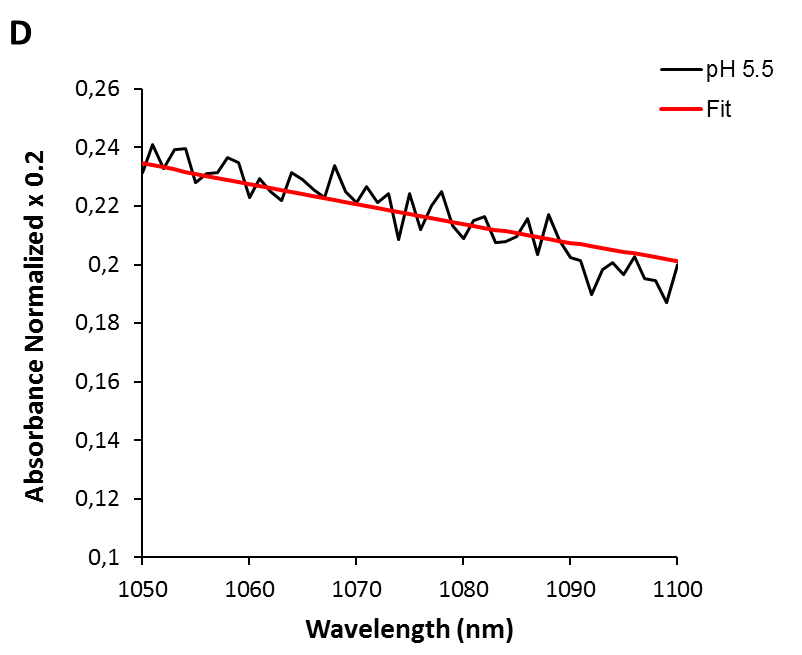


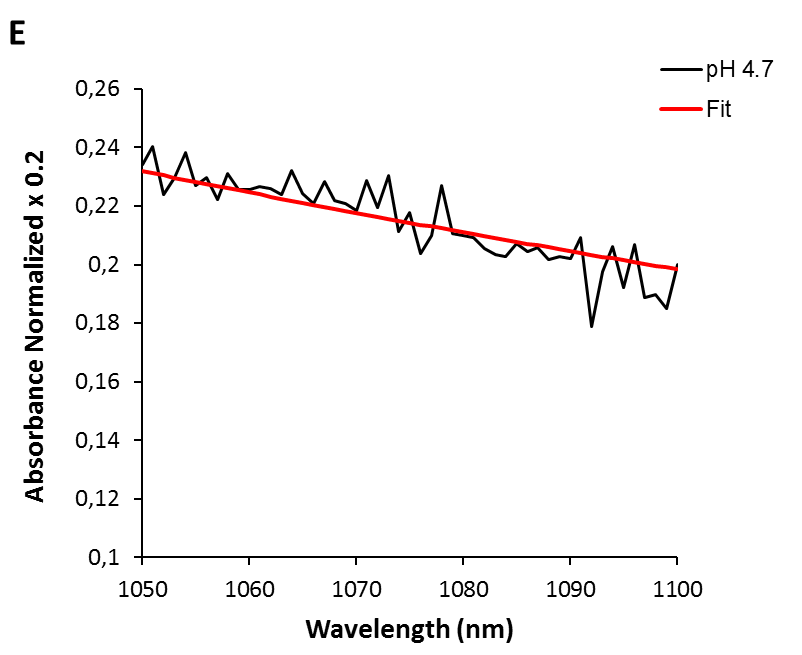

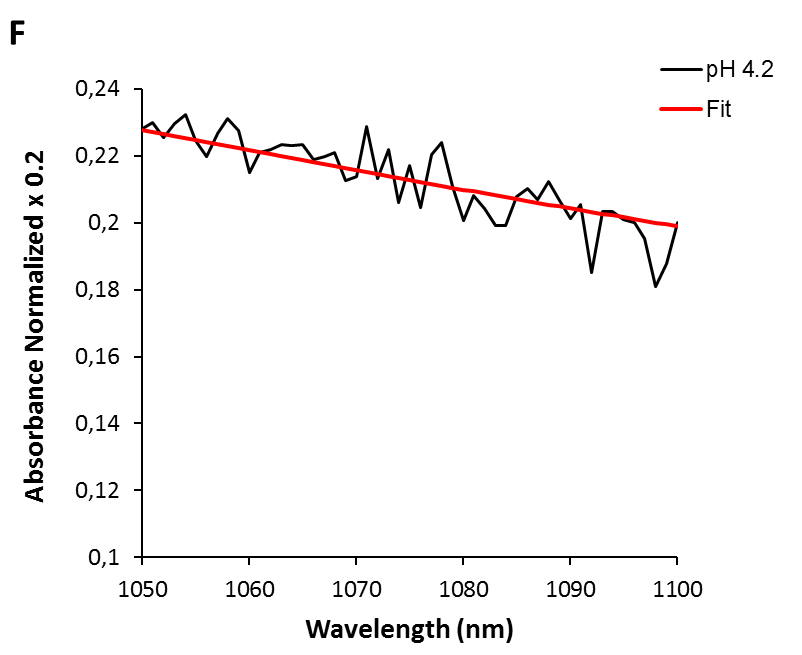


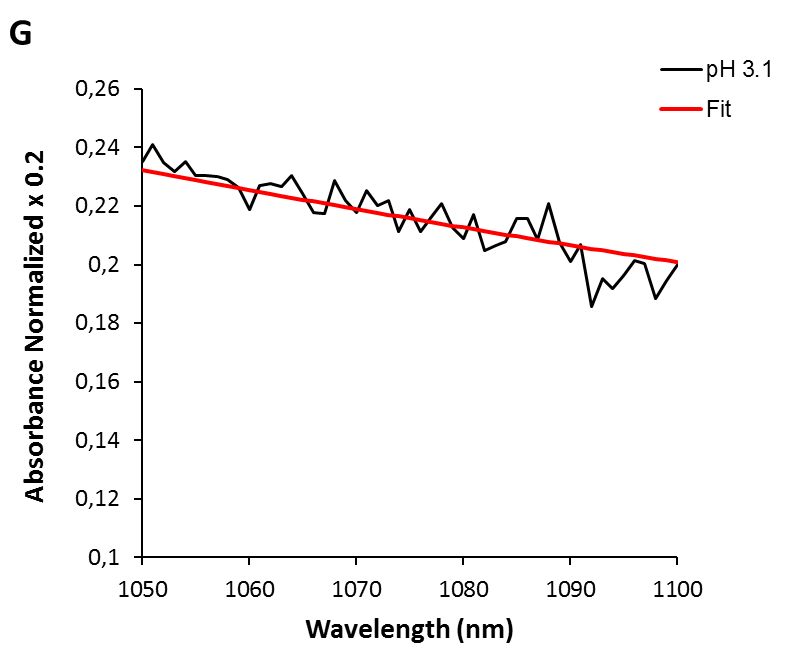

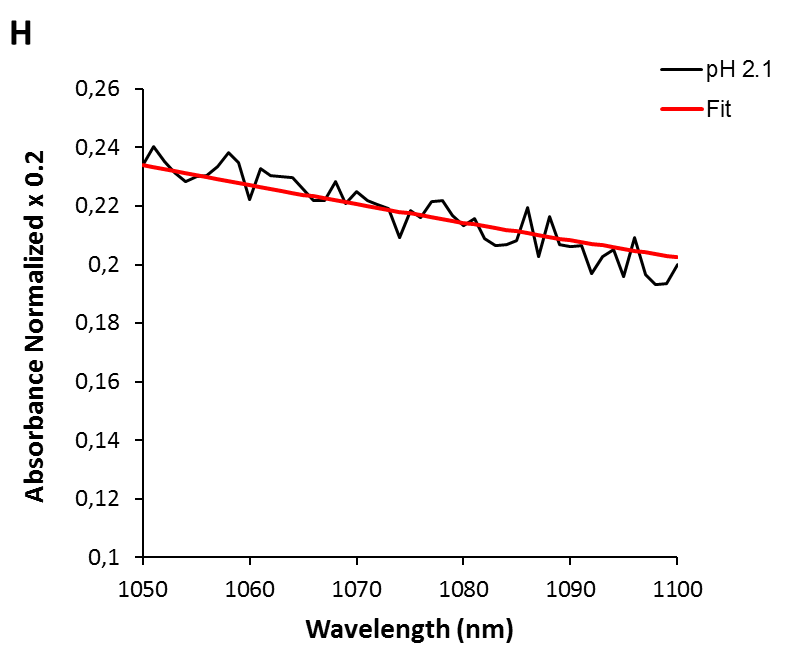


**Figure S3.** Application of Eq. 2 to the NIR spectra in the wavelength region from 1050 to 1100 nm of the Syn protein while varying the pH of the protein solution. (a–h) The normalized NIR spectra displayed in Figure 2b and the corresponding fits, for α-syn protein solutions at pH 7.0 to 2.1.


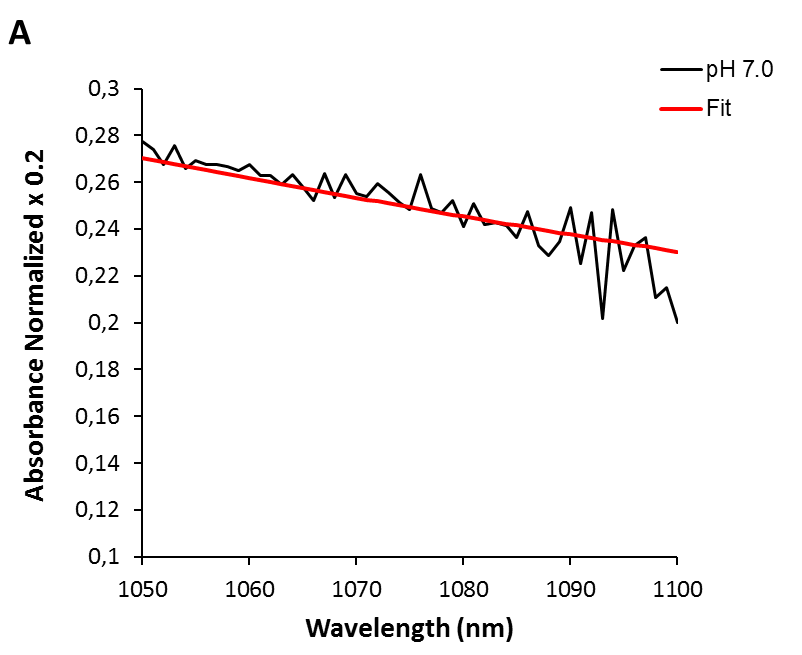

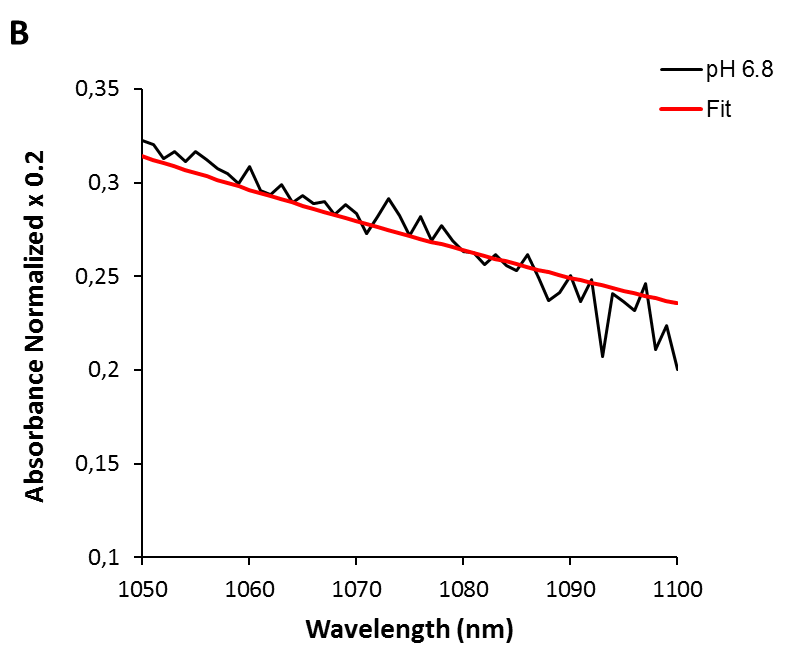


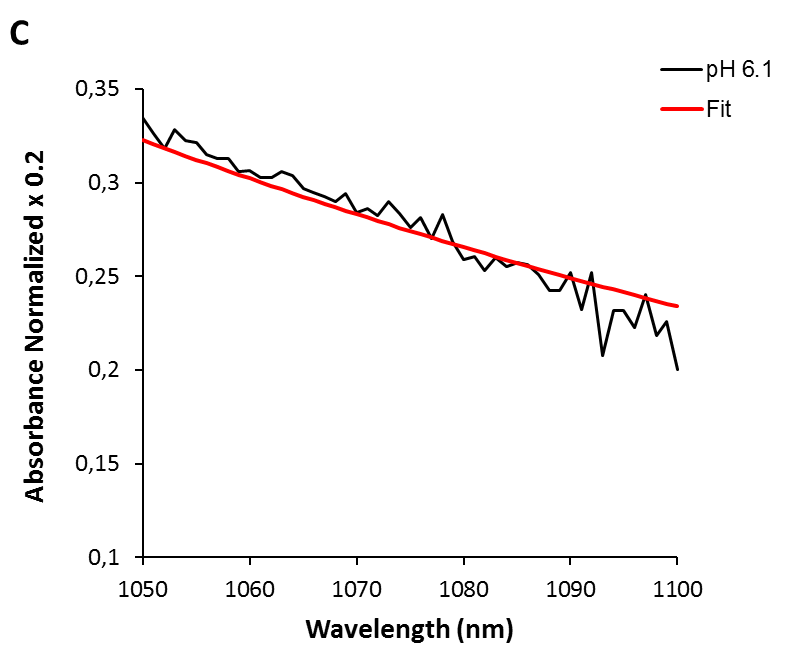

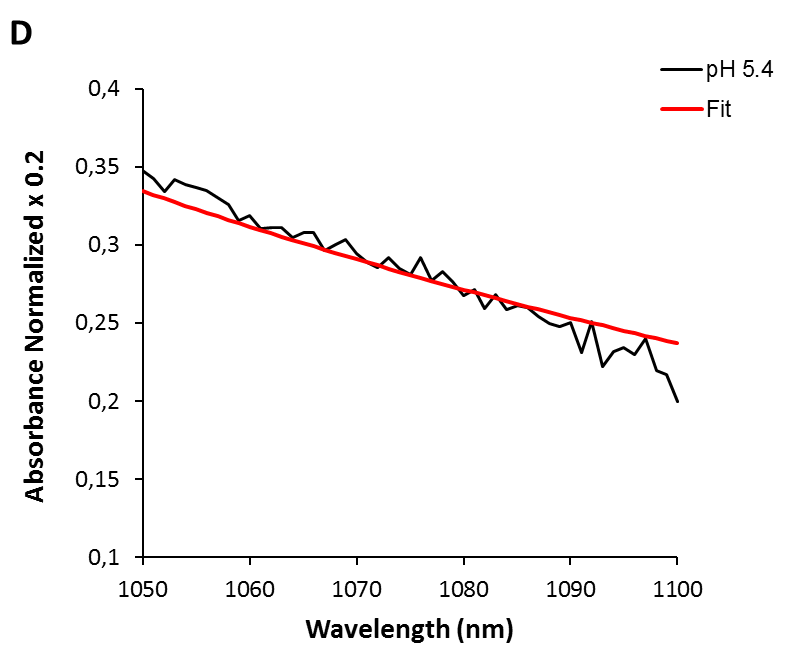


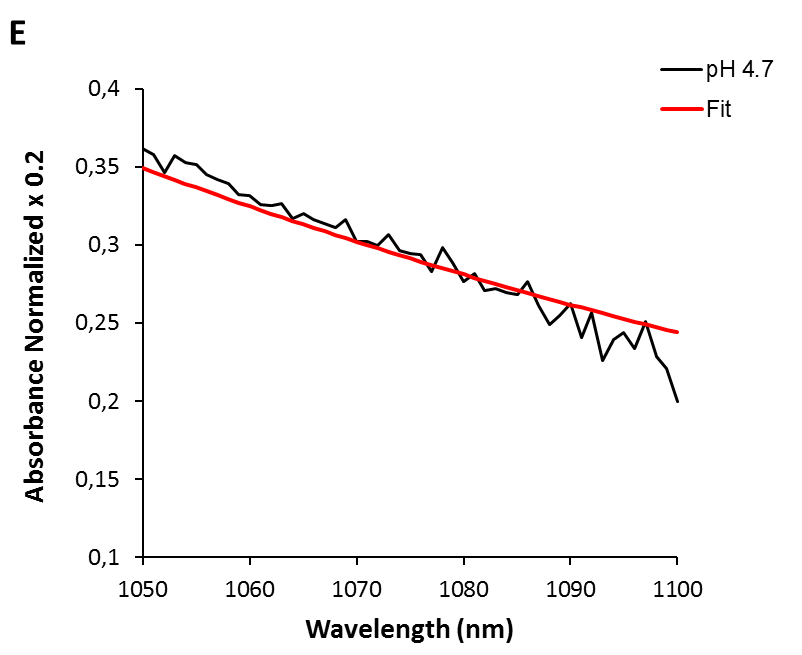

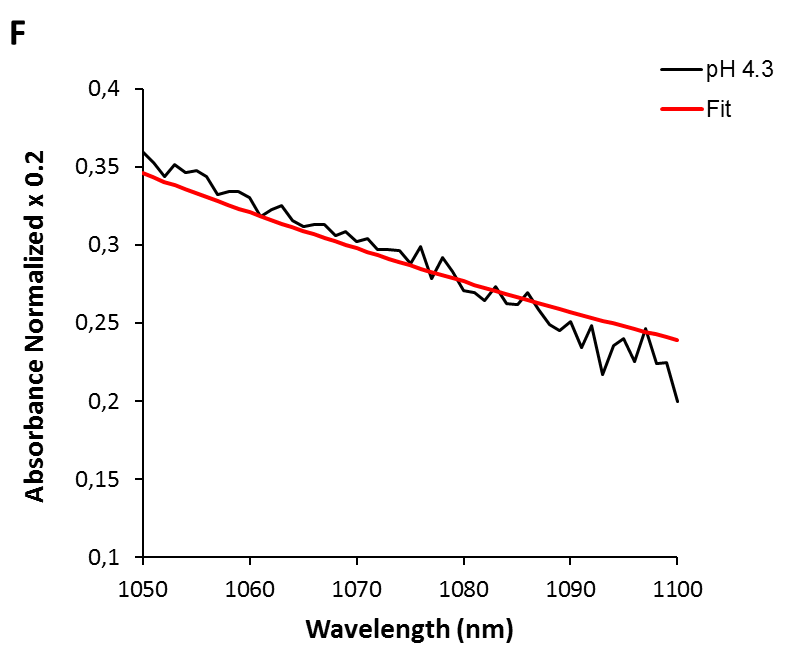


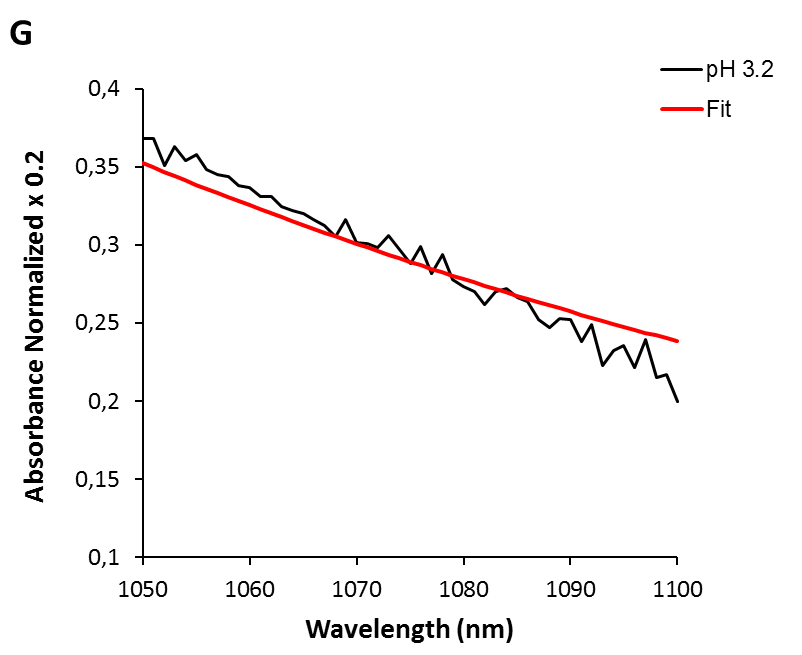

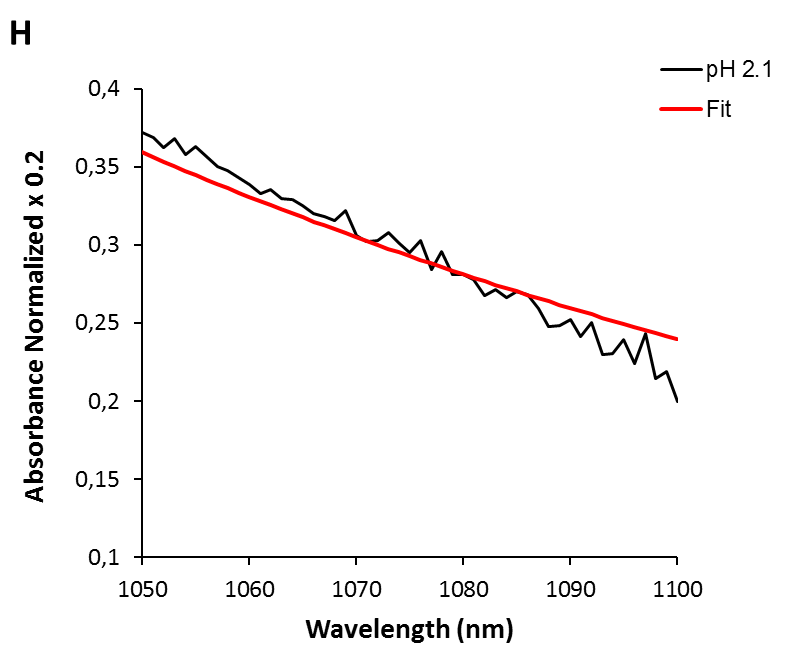


**Figure S4.** Application of Eq. 2 to the near-infrared spectra in the wavelength region from 1050 to 1100 nm of the Syn protein while varying the pH of the protein solution. (a–h) The normalized NIR spectra displayed in Figure 2c and the corresponding fits, for α-syn protein solutions at pH 7.0 to 2.1.

**Table S1.** Parameters calculated from the application of Eq. 2 (*n*, δ*n*, *k*, δ*k*, ln(*k*), and δln*k*)) to the NIR spectra in Figure 2a in the wavelength region from 1050 to 1100 nm. The χ^2^ values are also indicated.

|  |  | **δ(*n*) *=*** |  |  | **δln(*k*) *=*** | **χ^2^ (Fit)** |
| --- | --- | --- | --- | --- | --- | --- |
| **pH** | ***n*** | ***n_i + 1_* – *n_i_*** | ***k*** | **ln(*k*)** | **ln(*k*)*_i + 1_* – ln(*k*)*_i_*** |  |
| **7.0** | 0 | − | 0.1967 | −1.626 | − | 0.005 |
| **6.5** | −0.5146 | −0.5146 | 7.397 | 2.001 | 3.627 | 0.010 |
| **6.2** | −0.5958 | −0.0812 | 13.09 | 2.572 | 0.5708 | 0.007 |
| **5.8** | −0.4511 | 0.1447 | 4.547 | 1.514 | −1.057 | 0.006 |
| **5.2** | −0.6045 | −0.1534 | 14.10 | 2.646 | 1.132 | 0.007 |
| **4.4** | −0.9380 | −0.3335 | 143.7 | 4.968 | 2.322 | 0.006 |
| **3.1** | −0.5927 | 0.3452 | 13.22 | 2.582 | −2.386 | 0.009 |
| **2.1** | −0.1219 | 0.4708 | 0.4783 | −0.7376 | −3.319 | 0.017 |

**Table S2.** Parameters calculated from the application of Eq. 2 (*n*, δ*n*, *k*, δ*k*, ln(*k*), and δln(*k*)) to the NIR spectra in Figure 2b in the wavelength region from 1050 to 1100 nm. The χ^2^ values are also indicated.

|  |  | **δ(*n*) *=*** |  |  | **δln(*k*) *=*** | **χ^2^ (Fit)** |
| --- | --- | --- | --- | --- | --- | --- |
| **pH** | ***n*** | ***n_i + 1_* – *n_i_*** | ***k*** | **ln(*k*)** | **ln(*k*)*_i + 1_* – ln(*k*)*_i_*** |  |
| **7.0** | −0.0054 | − | 0.207 | −1.574 | − | 0.001 |
| **6.6** | −2.9178 | −2.9124 | 1.50 × 10^8^ | 18.83 | 15.93 | 0.008 |
| **6.2** | −3.1316 | −0.2138 | 6.57 × 10^8^ | 20.30 | 1.475 | 0.011 |
| **5.5** | −3.2931 | −0.1614 | 2.09 × 10^9^ | 21.46 | 1.155 | 0.009 |
| **4.7** | −3.3488 | −0.0557 | 3.04 × 10^9^ | 21.83 | 0.375 | 0.012 |
| **4.2** | −2.9112 | 0.4376 | 1.42 × 10^8^ | 18.77 | −3.061 | 0.010 |
| **3.1** | −3.1330 | −0.2217 | 6.78 × 10^8^ | 20.33 | 1.562 | 0.009 |
| **2.1** | −3.1166 | 0.0163 | 6.10 × 10^8^ | 20.23 | −0.106 | 0.006 |

**Table S3.** Parameters calculated from the application of Eq. 2 (*n*, δ*n*, *k*, δ*k*, ln(*k*), and δln(*k*)) to the NIR spectra in Figure 2c in the wavelength region from 1050 to 1100 nm. The χ^2^ values are also indicated.

|  |  | **δ(*n*) *=*** |  |  | **δln(*k*) *=*** | **χ^2^ (Fit)** |
| --- | --- | --- | --- | --- | --- | --- |
| **pH** | ***n*** | ***n_i + 1_* – *n_i_*** | ***k*** | **ln(*k*)** | **ln(*k*)*_i + 1_* – ln(*k*)*_i_*** |  |
| **7.0** | −3.4433 | − | 6.84 × 10^9^ | 22.65 | − | 0.019 |
| **6.8** | −5.8963 | −2.4530 | 1.54 × 10^17^ | 39.57 | 16.93 | 0.023 |
| **6.1** | −6.9194 | −1.0230 | 2.59 × 10^20^ | 47.00 | 7.431 | 0.028 |
| **5.4** | −7.4149 | −0.4956 | 8.44 × 10^21^ | 50.49 | 3.483 | 0.024 |
| **4.7** | −7.7275 | −0.3126 | 7.76 × 10^22^ | 52.71 | 2.218 | 0.028 |
| **4.3** | −7.9256 | −0.1981 | 3.05 × 10^23^ | 54.07 | 1.367 | 0.028 |
| **3.2** | −8.5874 | −0.6618 | 3.11 × 10^25^ | 58.70 | 4.628 | 0.033 |
| **2.1** | −8.8121 | −0.2246 | 1.51 × 10^26^ | 60.28 | 1.580 | 0.031 |

**Table S4.** Calculated particle radius (µm) and δ(radius) due to application of the third-order polynomial function of *n* (Table S1) as a function of the radius *r* (Eq. 5), yielding the particle radius of the homogeneous sphere, empirically.^1^

| **pH** | **Radius**  **(µm)** | **δ(Radius) =**  **Radius*_i + 1_* – Radius*_i_*** |
| --- | --- | --- |
| **7.0** | 0.785 | − |
| **6.5** | 0.497 | −0.288 |
| **6.2** | 0.386 | −0.111 |
| **5.8** | 0.630 | 0.244 |
| **5.2** | 0.380 | −0.250 |
| **4.4** | 0.269 | −0.111 |
| **3.1** | 0.388 | 0.119 |
| **2.1** | 0.760 | 0.372 |

**Table S5.** Calculated particle radius (µm) and δ(radius) due to application of the third-order polynomial functions of *n* (Table S2) as a function of the radius *r* (Eqs. 4 and 5), yielding the particle radius of the homogeneous sphere empirically.^1^

| **pH** | **Radius**  **(µm)** | **δ(Radius) =**  **Radius*_i + 1_* – Radius*_i_*** |
| --- | --- | --- |
| **7.0** | 0.784 | − |
| **6.6** | 0.086 | −0.698 |
| **6.2** | 0.077 | −0.009 |
| **5.5** | 0.070 | −0.007 |
| **4.7** | 0.068 | −0.002 |
| **4.2** | 0.086 | 0.018 |
| **3.1** | 0.077 | −0.009 |
| **2.1** | 0.078 | 0.001 |

**References**

1. A.M.K. Nilsson, C. Sturesson, D.L. Liu, S. Andersson-Engels. “Changes in Spectral Shape of Tissue Optical Properties in Conjunction with Laser-Induced Thermotherapy”. Appl. Opt. 1998. 37(7): 1256-1267. 10.1364/AO.37.001256
